# Inadequate use of antibiotics in the covid-19 era: effectiveness of antibiotic therapy

**DOI:** 10.1186/s12879-021-06821-1

**Published:** 2021-11-08

**Authors:** Alejandro David Bendala Estrada, Jorge Calderón Parra, Eduardo Fernández Carracedo, Antonio Muiño Míguez, Antonio Ramos Martínez, Elena Muñez Rubio, Manuel Rubio-Rivas, Paloma Agudo, Francisco Arnalich Fernández, Vicente Estrada Perez, María Luisa Taboada Martínez, Anxela Crestelo Vieitez, Paula Maria Pesqueira Fontan, Marta Bustamante, Santiago J. Freire, Isabel Oriol-Bermúdez, Arturo Artero, Julián Olalla Sierra, María Areses Manrique, H. Francisco Javier Carrasco-Sánchez, Vanessa Carolina Vento, Gema María García García, Pablo Cubero-Morais, José-Manuel Casas-Rojo, Jesús Millán Núñez-Cortés

**Affiliations:** 1 Internal Medicine Department, Gregorio Marañón General University Hospital, Madrid, Spain; 2 Internal Medicine Department, Puerta de Hierro Majadahonda University Hospital, Madrid, Spain; 3 Internal Medicine Department, Bellvitge University Hospital-IDIBELL, L’Hospitalet de Llobregat, Barcelona, Spain; 4 Internal Medicine Department, 12 de Octubre University Hospital, Madrid, Spain; 5 Internal Medicine Department, La Paz University Hospital, Madrid, Spain; 6 Internal Medicine Department, San Carlos Clinical Hospital, Madrid, Spain; 7 Internal Medicine Department, Cabueñes University Hospital, Gijón, Asturias Spain; 8 Internal Medicine Department, Royo Villanova Hospital, Zaragoza, Spain; 9 Internal Medicine Department, Santiago Clinical Hospital, Santiago de Compostela, A Coruña Spain; 10 Internal Medicine Department, La Princesa University Hospital, Madrid, Spain; 11 Internal Medicine Department, A Coruña University Hospital, A Coruña, Spain; 12 Infectious Diseases, Internal Medicine Department, Moisès Broggi Hospital, Sant Joan Despí, Barcelona Spain; 13 Internal Medicine Department, Dr. Peset University Hospital, Valencia, Spain; 14 Internal Medicine Department, Costa del Sol Hospital, Marbella, Málaga Spain; 15 Internal Medicine Department, Santa Marina Hospital, Bilbao, Spain; 16 Internal Medicine Department, Juan Ramón Jiménez University Hospital, Huelva, Spain; 17 Internal Medicine Department, Henares Hospital, Coslada, Madrid Spain; 18 Internal Medicine Department, Badajoz University Hospital Complex, Badajoz, Spain; 19 Internal Medicine Department, Río Hortega University Hospital, Regional Health Management of Castilla y Leon (SACYL), Valladolid, Spain; 20 Internal Medicine Department, Infanta Cristina University Hospital, Parla, Madrid Spain

**Keywords:** COVID-19, SARS-CoV-2, Antibiotics, Survival, Macrolides, Azithromycin

## Abstract

**Background:**

Since December 2019, the COVID-19 pandemic has changed the concept of medicine. This work aims to analyze the use of antibiotics in patients admitted to the hospital due to SARS-CoV-2 infection.

**Methods:**

This work analyzes the use and effectiveness of antibiotics in hospitalized patients with COVID-19 based on data from the SEMI-COVID-19 registry, an initiative to generate knowledge about this disease using data from electronic medical records. Our primary endpoint was all-cause in-hospital mortality according to antibiotic use. The secondary endpoint was the effect of macrolides on mortality.

**Results:**

Of 13,932 patients, antibiotics were used in 12,238. The overall death rate was 20.7% and higher among those taking antibiotics (87.8%). Higher mortality was observed with use of all antibiotics (OR 1.40, 95% CI 1.21–1.62; *p* < .001) except macrolides, which had a higher survival rate (OR 0.70, 95% CI 0.64–0.76; *p* < .001). The decision to start antibiotics was influenced by presence of increased inflammatory markers and any kind of infiltrate on an x-ray. Patients receiving antibiotics required respiratory support and were transferred to intensive care units more often.

**Conclusions:**

Bacterial co-infection was uncommon among COVID-19 patients, yet use of antibiotics was high. There is insufficient evidence to support widespread use of empiric antibiotics in these patients. Most may not require empiric treatment and if they do, there is promising evidence regarding azithromycin as a potential COVID-19 treatment.

**Supplementary Information:**

The online version contains supplementary material available at 10.1186/s12879-021-06821-1.

## Introduction—Background

In late December 2019, a series of pneumonia cases of an unknown etiology were diagnosed in Wuhan, Hubei province (China). One week later, a new betacoronavirus was identified and named severe acute respiratory syndrome coronavirus 2 (SARS-CoV-2), the virus that causes coronavirus disease 2019 (COVID-19) [1, 2]. In March 2020, this new disease was declared a pandemic by the World Health Organization (WHO) and as of May 31st, 2021, more than 169 million cases of COVID-19 and more than 3,500,000 deaths from it had been reported globally. Spain in particular has been one of the countries most affected by the COVID-19 pandemic, with more than 3,500,000 cases and 79,000 deaths as of that date [3–5]. Other most hitted countries by COVID-19 are India, United States and Brazil [6, 7].

Currently, in spring 2021, the available knowledge on how to manage patients with COVID-19 is incomplete and highly fragmented [8]. The U.S. Food and Drug Administration (FDA) has approved few drugs for treating the disease as Remdesivir. Nevertheless, physicians are using drugs approved for other indications while others are being studied. In this context, this work reflects on how to approach the challenge of treating this illness, particularly in regard to the use of antibiotics [9, 10].

The etiology of community-acquired pneumonia among hospitalized adults is unknown in 62% of cases, viral in 27% of cases, and bacterial in 14% of cases. Prior to December 2019, coronaviruses were responsible for 10% of viral pneumonias (2.7% of all etiologies) [11]. In lower respiratory tract infections, viruses can induce structural changes as reduction of ciliary function and decrease epithelial barrier function that can favor bacterial infections. It is not clear if antibiotics are necessary for these viral pneumonias [12–14]. Treatment guidelines for community-acquired pneumonia recommend initial empiric antibiotic therapy for possible bacterial infection or co-infection, given that they often coexist and there are no clear diagnostic tests for determining if the pneumonia is solely due to a virus at the time of onset [15, 16]. On the other hand, treatment decisions must be weighed taking into consideration the rise of multidrug-resistant bacteria and the fact that patients can develop complications associated with antibiotic use [17, 18].

Currently, there are no clear estimates on the incidence of bacterial co-infection in patients with COVID-19 and no clinical trials have been conducted on the use of empiric antibiotics in these patients [9]. Fluoroquinolones, such as ciprofloxacin and moxifloxacin, have been analyzed for their potential capacity to bind to the SARS-CoV-2 protease Mpro, blocking replication [19]. Furthermore, beta-lactam antibiotics are being evaluated in critically ill patients with SARS-CoV-2 infection, but more clinical trials are necessary in order to properly evaluate results [20].

Some researchers have concentrated on the use of macrolides in patients with COVID-19. Some macrolides, such as azithromycin and clarithromycin, are being studied not only for their anti-bacterial activity, but also their immunomodulatory and anti-inflammatory effects. They could be particularly useful in viral infections such as COVID-19, which are associated with an excessive inflammatory response, through the antibiotics’ attenuation of cytokine production [21–23]. Likewise, azithromycin has shown effects against virus replication and internalization processes in other viruses such as influenza A virus subtype H1N1 or Zika virus [24, 25].

With this background, this work aims to analyze the use of antibiotics in patients admitted to the hospital due to SARS-CoV-2 infection.

## Methods

This work is a multicenter, nationwide, observational study based on patient data obtained from the SEMI-COVID-19 Registry.

### Study design and population

The SEMI-COVID-19 Registry is an enterprise of the Spanish Society of Internal Medicine (SEMI, for its initials in Spanish) to advance knowledge on the epidemiology, clinical progress, risk factors, complications of patients infected with SARS-CoV-2 with the aim of improving SARS-CoV-2 treatment. The list of SEMI-COVID-19 Registry members can be found in Additional file 1.

**Fig. 1:**
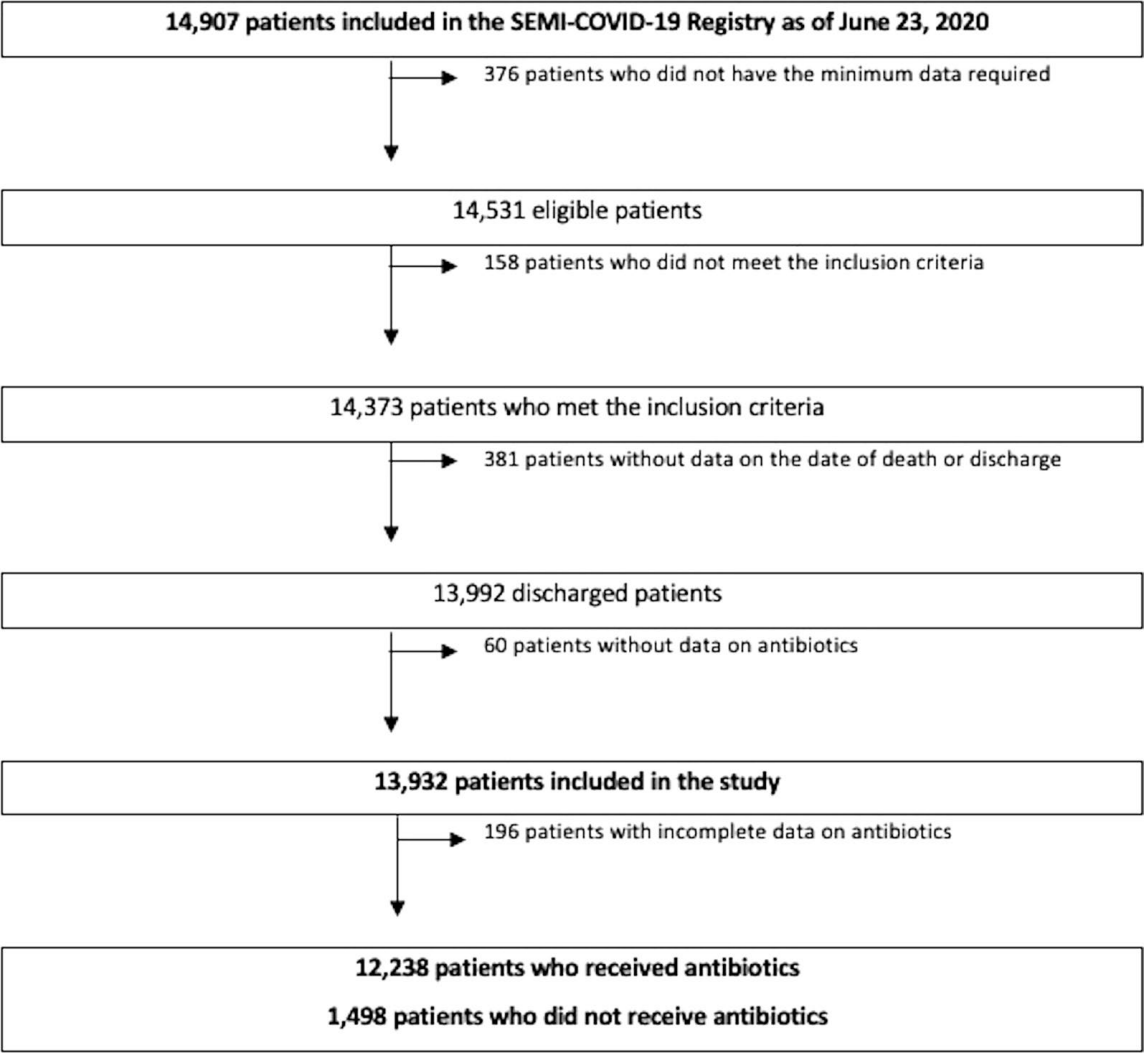
Patient inclusion flow chart

Informed consent was obtained from all participants for using of their medical data for all research derived from the SEMI-COVID-19 registry. The registry is an anonymized online database of retrospective data on consecutive adult patients with COVID-19 hospitalized in internal medicine departments throughout Spain from March 1 to May 23, 2020. The diagnosis was confirmed microbiologically by real time transcription polymerase chain reaction (Rt-PCR) testing of a nasopharyngeal sample. Exclusion criteria were subsequent admissions of the same patient and denial or withdrawal of informed consent. Patients were cared for at their attending physician’s discretion, according to local protocols and their clinical judgment. Patient inclusion flow chart is shown in Fig. 1.

The registry includes data on more than 300 variables in categories such as:

- Sociodemographic and epidemiological data
- Personal medical and medication history
- Symptoms and physical examination findings upon admission
- Laboratory test results
- Radiological findings and their progress
- Pharmacological treatment and ventilatory support
- In-hospital complications and causes of death

More in-depth information on the registry and preliminary results are available in a previously published work [4].

The SEMI-COVID-19 Registry was approved by the Provincial Research Ethics Committee of Málaga (Spain).

### Study conclusion

The primary endpoint was all-cause in-hospital mortality according to use of antibiotic therapy. The secondary endpoint was the specific effect of macrolides on the all-cause mortality rate. The follow-up period was from admission to discharge or death, including early readmissions.

We have analyzed the criteria for the use of antibiotics, any relationship to epidemiological or microbiological factors, and the evolution of analytical and radiological parameters.

### Literature search

A literature search was conducting using the MEDLINE database with the following search terms: “antibiotics and COVID-19,” “bacterial co-infections and SARS-CoV-2,” and “azithromycin and COVID-19.” The most up-to-date evidence and all information regarding antibiotics, macrolides, and bacterial co-infections in COVID-19 reported in English or Spanish were selected.

### Data analysis

The patients were initially divided into two groups according to use of antibiotic therapy. The first group, which included 12,238 patients, received antibiotics and the second, with 1498 patients, did not receive antibiotics.

Continuous quantitative variables were tested for normal distribution using rates of skewness and kurtosis, Levene’s test, or the Kolmogorov–Smirnov test, as appropriate. These variables are expressed as medians and interquartile range (IQR). Comparisons between groups were made using the Student’s T-test, Mann–Whitney U test, Wilcoxon test, analysis of variance (ANOVA), or the Kruskal–Wallis test. Categorical variables are expressed absolute values and percentages. Differences in proportions were analyzed using the Chi-square test, McNemar’s test, or Fisher’s exact test, as appropriate.

We also used a bivariate logistic regression to evaluate the relationship between groups of antibiotics and mortality. A multivariate analysis was carried out to correct for confounding variables using clinically relevant, statistically significant variables (*p* < 0.001) identified in the univariate analysis.

Measures of association are expressed as odds ratio (OR) with 95% confidence intervals (95% CI). Statistical analysis was carried out using STATA software (v14.2). Statistical significance was established as *p* < 0.05.

## Results

### Demographics, mortality, and clinical features

Patients were initially divided into two groups according to whether they received antibiotic therapy or not. Of a total of 13,932 patients included in this study, antibiotics were used in 12,238 (87.8%) and not used in 1498 (10.8%). A higher mortality rate was observed with the use of all antibiotics except macrolides, which showed a higher survival rate (OR 0.70, 95% CI 0.64–0.76; *p* < 0.001). Tables 1 and 2 show the type of antibiotic used and the number of patients who died or survived. Microbiological findings are shown on Table 3.

**Table 1:** Use of antibiotic therapy in COVID-19 patients admitted to internal medicine departments

Antibiotic used	No. (Total n = 13,932) (%)
Any antibiotic	12,238 (87.8)
Beta-lactams	10,031 (72.0)
Macrolides	8382 (60.2)
Quinolones	1850 (13.3)

*It was possible for a patient to receive more than one antibiotic concomitantly*

**Table 2:** Antibiotic used and relationship to mortality

Antibiotic used	Overall (n = 13,932) (%)	Survivors n = 11,042 (%)	Deceased n = 2890 (%)	Odds ratio (95% CI)	*p* value
Any antibiotic	12,238 (87.8)	9641 (88.5)	2597 (91.4)	1.39 (1.20–1.61)	< 0.001
Beta-lactams	10,031 (72.0)	7709 (70.0)	2322 (80.5)	1.77 (1.60–1.96)	< 0.001
Macrolides	8382 (60.2)	6845 (62.2)	1537 (53.5)	0.70 (0.64–0.76)	< 0.001
Quinolones	1850 (13.3)	1363 (12.5)	487 (17.1)	1.44 (1.29–1.62)	< 0.001

**Table 3:** Microbiological findings—SARS-CoV-2 infection

	No. (Total n = 13,932)	No. (%)
Confirmed COVID-19	13,932	13,932 (100.0)
Acquisition of COVID-19		
Community	13,870	11,806 (85.1)
Nosocomial		908 (6.6)
Nursing home		1156 (8.3)
Source of positive sample for SARS-CoV-2		
Nasopharyngeal swab	13,672	13,396 (98.0)
Sputum		224 (1.6)
Bronchoalveolar lavage (BAL)		52 (0.4)
Results of the first PCR		
Negative	13,723	1660 (12.1)
Positive		12,063 (87.9)
Results of urine antigens		
Negative	13,570	6168 (45.5)
Any positive		198 (1.5)
Positive Pneumococcus		179 (1.3)
Positive Legionella		12 (0.1)
Both positive		7 (0.1)
Not performed		7204 (53.1)
HIV serology test		
Not performed	13,793	5860 (42.5)
Negative		7844 (56.9)
Positive		89 (0.7)

Differences in fatality have been noted according to where the virus was acquired: mortality was higher among those who acquired the infection nosocomially (OR 1.98, 95% CI 1.71–2.30; *p* < 0.001) or in a nursing home (OR 2.80, 95% CI 2.46–3.18; *p* < 0.001) compared to those who were infected in the community (Table 4). Differences regarding the use of antibiotics and macrolides in particular according to where the infection was contracted are shown in Tables 5 and 6. Multivariate analyses of mortality based on the use of antibiotics and specifically on the use of macrolides were carried out with the possible confounding variables of age, degree of dependence, and place of disease acquisition. The results are shown in Tables 7 and 8.

**Table 4:** Mcrobiological findings and relationship to mortality

	Total (n = 13,932)	No. (%)	Survivors n = 11,042 (%)	Deceased n = 2890 (%)	Odds ratio (95% CI)	*p* value
Acquisition of COVID-19						
Community	13,870	11,806 (85.1)	9653 (87.8)	2153 (74.9)	1. (ref)	–
Nosocomial		908 (6.6)	630 (5.7)	278 (9.7)	1.98 (1.71–2.30)	< 0.001
Nursing Home		1156 (8.3)	712 (6.5)	444 (15.4)	2.80 (2.46–3.18)	< 0.001
Results of urine antigens						
Negatives	13,570	6168 (45.5)	5086 (47.3)	1082 (38.5)	1. (ref)	–
Any positive		198 (1.5)	146 (1.4)	52 (1.9)	1.67 (1.21–2.31)	0.002
Not performed		7204 (53.1)	5529 (51.4)	1675 (59.6)	1.42 (1.31–1.55)	< 0.001

**Table 5:** Microbiological findings according to use of antibiotics

	Total (n = 13,932)	No. (%)	With antibiotics n = 12,238 (%)	Without antibiotics n = 1498 (%)	*p* value
Acquisition of COVID-19					
Community	13,674	11,633 (85.1)	10,465 (85.9)	1168 (78.7)	< 0.001
Nosocomial		891 (6.5)	760 (6.2)	131 (8.8)	
Nursing Home		1150 (8.4)	965 (7.9)	185 (12.5)	
Results of urine antigens					
Negative	13,381	6077 (45.4)	5569 (46.6)	508 (35.6)	< 0.001
Any positive		196 (1.5)	189 (1.6)	7 (0.5)	
Not performed		7108 (53.1)	6195 (51.8)	913 (63.9)

**Table 6:** Microbiological findings according to use of macrolides

	Total (n = 13,932)	No. (%)	Macrolides n = 8382 (%)	Without macrolides n = 5502 (%)	p value
Acquisition of COVID-19					
Community	13,822	11,766 (85.1)	7315 (87.5)	4451 (81.5)	< 0.001
Nosocomial		903 (6.5)	460 (5.5)	443 (8.1)	
Nursing Home		1153 (8.3)	588 (7.0)	565 (10.4)	
Results of urine antigens					
Negative	13,523	6147 (45.5)	4124 (50.1)	2023 (38.2)	< 0.001
Any Positive		198 (1.5)	141 (1.7)	57 (1.1)	
Not performed		7178 (53.1)	3962 (48.2)	3216 (60.7)

**Table 7:** Antibiotic therapy used and relationship to mortality (Multivariate analysis adjusted according to patient age and frailty)

	Odds ratio (95% CI)	*p* value
Use of antibiotic therapy	1.52 (1.29–1.80)	< 0.001
Age	1.08 (1.08–1.09)	< 0.001
Degree of dependence		
Independent or mild	–	1 (ref.)
Moderate	1.78 (1.54–2.06)	< 0.001
Severe	2.05 (1.72–2.43)	< 0.001
Acquisition of COVID-19		
Community	–	1 (ref.)
Nosocomial	1.71 (1.43–2.04)	< 0.001
Nursing Home	0.66 (0.56–0.78)	< 0.001

**Table 8:** Use of macrolides and relationship to mortality  (Multivariate analysis adjusted according to patient age and frailty)

	Odds ratio (95% CI)	*p* value
Use of macrolides	0.80 (0.73–0.88)	< 0.001
Age	1.08 (1.08–10.9)	< 0.001
Degree of dependence		
Independent or mild	–	1 (ref.)
Moderate	1.80 (1.56–2.07)	< 0.001
Severe	2.02 (1.70–2.40)	< 0.001
Acquisition of COVID-19		
Community	–	1 (ref.)
Nosocomial	1.65 (1.38–1.97)	< 0.001
Nursing Home	0.62 (0.53–0.73)	< 0.001

Older age was a factor that differed between those who received antibiotics versus those who did not in a significant manner (69 years [IQR 56–79] vs. 67 years [IQR 52–80]; *p* < 0.001). There was a lower rate of antibiotic use among patients with dementia (9.9% vs. 11.7%; *p* < 0.05), neurodegenerative disease (8.9% vs. 11.4%; *p* < 0.05), and moderate and severe dependence. This may be because we tend to be more cautious in the treatments applied to these groups of patients. Macrolides were more commonly used in men and in those between 40 and 80 years of age. They were less commonly used in patients with previous heart disease such as atrial fibrillation, myocardial infarction, or congestive heart failure. The demographic differences between groups that did and did not receive antibiotics and according to macrolide use are shown in Tables 9 and 10.

**Table 9:** Demographic data and comorbidities according to use of antibiotic therapy

	Total (n = 13,932)	No. (%)	With antibiotics n = 12,238 (%)	Without antibiotics n = 1498 (%)	*p* value
Median (IQR) age (years)		69 (56–80) [18–105]	69 (56–79)	67 (52–80)	< 0.001
Age (years)					
< 40 years	13,736	874 (6.4)	732 (6.0)	142 (9.5)	0.002
40–50 years		1338 (9.7)	1143 (9.3)	195 (13.0)	
50–60 years		2175 (15.8)	1955 (16.0)	220 (14.7)	
60–70 years		2686 (19.6)	2442 (20.0)	244 (16.3)	
70–80 years		3277 (23.9)	2965 (24.2)	312 (20.8)	
> 80 years		3386 (24.7)	3001 (24.5)	385 (25.7)	
Sex					
Women	13,721	5902 (43.0)	5137 (42.0)	765 (51.1)	< 0.001
Men		7819 (57.0)	7087 (58.0)	732 (48.9)	
Hypertension	13,714	6944 (50.6)	6261 (51.2)	683 (45.7)	< 0.001
Diabetes Mellitus	13,691	2617 (19.1)	2363 (19.4)	254 (17.1)	0.034
Dyslipidemia	13,708	5420 (39.5)	4888 (40.0)	532 (35.6)	0.001
Obesity (BMI > 30)	6,231	2102 (33.7)	1916 (33.9)	186 (31.8)	0.30
Smoking status					
Never	13,077	9130 (69.8)	8058 (69.2)	1072 (75.1)	< 0.001*
Former		3254 (24.9)	2995 (25.7)	259 (18.2)	
Current		693 (5.3)	597 (5.1)	96 (6.7)	
Alcohol use disorder	13,270	615 (4.6)	555 (4.7)	60 (4.1)	0.33
Atrial fibrillation	13,704	1535 (11.2)	1372 (11.2)	163 (10.9)	0.68
Myocardial infarction	13,703	1091 (8.0)	975 (8.0)	116 (7.8)	0.75
Congestive heart failure	13,708	975 (7.1)	850 (7.0)	125 (8.4)	0.048
Chronic pulmonary disease	13,710	942 (6.9)	849 (7.0)	93 (6.2)	0.29
Chronic bronchitis	13,708	694 (5.1)	627 (5.1)	67 (4.5)	0.28
Asthma	13,706	1002 (7.3)	888 (7.3)	114 (7.6)	0.63
Obstructive sleep apnea syndrome	13,643	832 (6.1)	756 (6.2)	76 (5.1)	0.09
Peripheral vascular disease	13,701	642 (4.7)	565 (4.6)	77 (5.2)	0.37
Dementia	13,708	1384 (10.1)	1209 (9.9)	175 (11.7)	0.029
Cerebrovascular disease	13,690	984 (7.2)	864 (7.1)	120 (8.0)	0.18
Hemiplegia	13,717	225 (1.6)	200 (1.6)	25 (1.7)	0.93
Neurodegenerative disease	13,713	1258 (9.2)	1087 (8.9)	171 (11.4)	0.001
Chronic kidney disease	13,704	821 (6.0)	746 (6.1)	75 (5.0)	0.09
Dialysis	13,678	138 (1.0)	123 (1.0)	15 (1.0)	0.29
Chronic liver disease	13,675	505 (3.7)	451 (3.7)	54 (3.6)	0.89
Cancer	13,694	1113 (8.1)	984 (8.1)	129 (8.6)	0.46
Solid metastatic tumor	13,704	283 (2.1)	248 (2.0)	35 (2.3)	0.43
Leukemia	13,716	167 (1.2)	157 (1.3)	10 (0.7)	0.040
Lymphoma	13,706	194 (1.4)	173 (1.4)	21 (1.4)	0.97
Peptic ulcer	13,700	350 (2.6)	310 (2.6)	40 (2.7)	0.76
Rare disease	13,673	278 (2.0)	248 (2.0)	30 (2.0)	0.95
Rheumatic disease	13,696	318 (2.3)	288 (2.4)	30 (2.0)	0.39
Organ transplantation	13,563	166 (1.2)	149 (1.2)	17 (1.2)	0.81
HIV infection	13,677	94 (0.7)	80 (0.7)	14 (0.9)	0.22
Acquired immunodeficiency syndrome (AIDS)	13,681	40 (0.3)	35 (0.3)	5 (0.3)	0.80
Degree of dependence					
Independent or mild	13,540	11,290 (83.4)	10,096 (83.7)	1194 (81.2)	0.010
Moderate		1273 (9.4)	1130 (9.4)	143 (9.7)	
Severe		977 (7.2)	843 (7.0)	134 (9.1)	
Charlson Comorbidity Index, median (IQR)	13,373	1 (0–2)	1 (0–2)	1 (0–2)	0.84
Age-adjusted Charlson Comorbidity Index, median (IQR)		3 (2–5)	3 (1–5)	3 (2–5)	0.08

*Mann–Whitney U test

**Table 10:** Demographic data and comorbidities according to use of  macrolides

	Total (n = 13,932)	No. (%)	With macrolides n = 8382 (%)	Without macrolides n = 5502 (%)	*p* value
Median (IQR) age (years)		69 (56–80) [18–105]	68 (56–79)	71 (57–82)	< 0.001
Age (years)					
< 40 years	13,884	882 (6.4)	483 (5.8)	399 (7.3)	< 0.001*
40–50 years		1359 (9.8)	843 (10.1)	516 (9.4)	
50–60 years		2199 (15.8)	1418 (16.9)	781 (14.2)	
60–70 years		2708 (19.5)	1756 (21.0)	952 (17.3)	
70–80 years		3318 (23.9)	2024 (24.15)	1294 (23.5)	
> 80 years		3418 (24.6)	1858 (22.2)	1560 (28.4)	
Sex					
Women	13,869	5953 (42.9)	3464 (41.4)	2489 (45.3)	< 0.001
Men		7916 (57.1)	4912 (58.6)	3004 (54.7)	
Hypertension	13,862	7010 (50.6)	4223 (50.5)	2787 (50.8)	0.74
Diabetes Mellitus	13,838	2645 (19.1)	1550 (18.5)	1095 (20.0)	0.033
Dyslipidemia	13,856	5479 (39.5)	3326 (39.8)	2153 (39.2)	0.53
Obesity (BMI > 30)	6287	2128 (33.9)	1387 (35.4)	741 (31.3)	0.001
Smoking status					
Never	13,214	9212 (69.7)	5522 (68.9)	3690 (71.0)	0.021
Former		3299 (25.0)	2083 (26.0)	1216 (23.4)	
Current		703 (5.3)	413 (5.2)	290 (5.6)	
Alcohol use disorder	13,412	624 (4.7)	380 (4.7)	244 (4.6)	0.82
Atrial fibrillation	13,851	1552 (11.2)	837 (10.0)	715 (13.0)	< 0.001
Myocardial infarction	13,849	1103 (8.0)	625 (7.5)	478 (8.7)	0.009
Congestive heart failure	13,855	988 (7.1)	518 (6.2)	470 (8.6)	< 0.001
Chronic pulmonary disease	13,856	948 (6.8)	519 (6.2)	429 (7.8)	< 0.001
Chronic bronchitis	13,855	703 (5.1)	424 (5.1)	279 (5.1)	0.96
Asthma	13,853	1010 (7.3)	623 (7.5)	387 (7.1)	0.38
Obstructive sleep apnea syndrome	13,791	846 (6.1)	549 (6.6)	297 (5.4)	0.006
Peripheral vascular disease	13,848	652 (4.7)	383 (4.6)	269 (4.9)	0.39
Dementia	13,852	1392 (10.1)	691 (8.3)	701 (12.8)	< 0.001
Cerebrovascular disease	13,837	994 (7.2)	553 (6.6)	441 (8.0)	0.002
Hemiplegia	13,863	228 (1.6)	119 (1.4)	109 (2.0)	0.011
Neurodegenerative disease	13,860	1268 (9.2)	610 (7.3)	658 (12.0)	< 0.001
Chronic kidney disease	13,851	828 (6.0)	493 (5.9)	335 (6.1)	0.62
Dialysis	13,826	140 (1.0)	78 (0.9)	62 (1.1)	0.011
Chronic liver disease	13,821	511 (3.7)	298 (3.6)	213 (3.9)	0.33
Cancer	13,842	1128 (8.2)	602 (7.2)	526 (9.6)	< 0.001
Solid metastatic tumor	13,852	284 (2.1)	147 (1.8)	137 (2.5)	0.003
Leukemia	13,864	169 (1.2)	107 (1.3)	62 (1.1)	0.43
Lymphoma	13,854	198 (1.4)	94 (1.1)	104 (1.9)	< 0.001
Peptic ulcer	13,848	353 (2.6)	208 (2.5)	145 (2.6)	0.58
Rare disease	13,821	280 (2.0)	133 (1.6)	147 (2.7)	< 0.001
Rheumatic disease	13,844	321 (2.3)	184 (2.2)	137 (2.5)	0.26
Organ transplantation	13,708	170 (1.2)	97 (1.2)	73 (1.4)	0.37
HIV infection	13,825	97 (0.7)	55 (0.7)	42 (0.8)	0.46
Acquired immunodeficiency syndrome (AIDS)	13,828	40 (0.3)	26 (0.3)	14 (0.3)	0.55
Degree of dependence					
Independent or mild	13,680	11,415 (83.4)	7093 (85.8)	4322 (79.9)	< 0.001
Moderate		1283 (9.4)	703 (8.5)	580 (10.7)	
Severe		982 (7.2)	473 (5.7)	509 (9.4)	
Charlson Comorbidity Index, median (IQR)	13,511	1 (0–2)	1 (0–2)	1 (0–2)	< 0.001
Age-adjusted Charlson Comorbidity Index, median (IQR)		3 (2–5)	3 (1–5)	4 (2–6)	< 0.001

*Mann–Whitney U test

Regarding patients’ previous treatment, a higher percentage of patients who were taking hydroxychloroquine received antibiotics (0.6% vs. 0.1%; *p* < 0.05). In the macrolide group, a lower percentage of patients were being treated with systemic corticosteroids (4% vs. 4.7%; *p* = 0.033) and biological therapies (1.1% vs. 1.6%; *p* = 0.016) (Tables 11 and 12).

**Table 11:** Use of antibiotic therapy according to habitual treatment

	Total (n = 13,932)	No. (%)	With antibiotics n = 12,238 (%)	Without antibiotics n = 1498 (%)	*p* value
Highly active antiretroviral therapy (HAART)	13,706	91 (0.7)	79 (0.7)	12 (0.8)	0.49
Metformin	13,713	1873 (13.7)	1690 (13.8)	183 (12.2)	0.09
Systemic corticosteroids	13,703	583 (4.3)	525 (4.3)	58 (3.9)	0.44
Inhaled corticosteroids	13,663	1296 (9.5)	1173 (9.6)	123 (8.3)	0.09
Hydroxychloroquine	13,707	69 (0.5)	67 (0.6)	2 (0.1)	0.032
Rapamycin (sirolimus)	13,675	62 (0.5)	57 (0.5)	5 (0.3)	0.68
Immunosuppressants	13,689	477 (3.5)	433 (3.6)	44 (3.0)	0.23
Biological therapy (monoclonal antibodies)	13,703	177 (1.3)	155 (1.3)	22 (1.5)	0.52

**Table 12:** Use of macrolides according to habitual treatment

	Total (n = 13,932)	No. (%)	With macrolides n = 8382 (%)	Without macrolides n = 5502 (%)	*p* value
Highly active antiretroviral therapy (HAART)	13,853	93 (0.7)	57 (0.7)	36 (0.7)	0.86
Metformin	13,860	1890 (13.6)	1154 (13.8)	736 (13.4)	0.51
Systemic corticosteroids	13,849	591 (4.3)	332 (4.0)	259 (4.7)	0.033
Inhaled corticosteroids	13,808	1304 (9.4)	796 (9.6)	508 (9.3)	0.61
Hydroxychloroquine	13,855	70 (0.5)	51 (0.6)	19 (0.4)	0.033
Rapamycin (sirolimus)	13,820	63 (0.5)	41 (0.5)	22 (0.4)	0.44
Immunosuppressants	13,836	486 (3.5)	278 (3.3)	208 (3.8)	0.14
Biological therapy (monoclonal antibodies)	13,851	180 (1.3)	93 (1.1)	87 (1.6)	0.016

In terms of patients’ clinical condition upon admission, the presence of fever (> 38 °C), cough, shortness of breath, arthralgia, fatigue, anorexia, and gastrointestinal symptoms were associated with an increased use of antibiotic therapy. Signs of general illness such as oxygen saturation < 90%, tachypnea, or tachycardia were also associated with increased rates of antibiotic use. Notably relevant is the presence of crackles on lung auscultation in up to 52.6% of patients. Like rhonchi (10.8% of patients), crackles were also associated with antibiotic use. All data on symptoms are shown in Table 13. Regarding the progression of respiratory parameters shown in Tables 14, 15, and 16, significant trends towards improvement were observed between the respiratory parameters on admission and those observed at 1 week in all patients.

**Table 13:** Use of antibiotic therapy according to initial clinical condition

	Total (n = 13,932)	No. (%)	With antibiotics n = 12,238 (%)	Without antibiotics n = 1498 (%)	*p* value
Symptoms					
Time from onset of symptoms, median (IQR)	13,576	6 (3–9)	7 (4–9)	6 (2–8)	< 0.001
Fever					
No (< 37 °C)	13,692	2137 (15.6)	1778 (14.6)	359 (24.0)	< 0.001
Low-grade fever (37–37.9 °C)		2865 (20.9)	2487 (20.4)	378 (25.3)	
Fever (> 38 °C)		8690 (63.5)	7932 (65.0)	758 (50.7)	
Shortness of breath	13,677	7879 (57.6)	7182 (58.9)	697 (46.8)	< 0.001
Sore throat	13,504	1294 (9.6)	1137 (9.5)	157 (10.6)	0.16
Cough					
No	13,689	3600 (26.3)	3106 (25.5)	494 (33.1)	< 0.001
Dry		7957 (58.1)	7132 (58.5)	825 (55.3)	
Productive		2132 (15.6)	1958 (16.1)	174 (11.7)	
Arthralgia	13,568	4073 (30.0)	3695 (30.6)	378 (25.6)	< 0.001
Fatigue	13,533	5875 (43.4)	5346 (44.4)	529 (35.7)	< 0.001
Anorexia	13,471	2634 (19.6)	2415 (20.1)	219 (14.8)	< 0.001
Ageusia	13,352	1002 (7.5)	910 (7.7)	92 (6.3)	0.06
Anosmia	13,345	892 (6.7)	804 (6.8)	88 (6.0)	0.27
Headache	13,516	1531 (11.3)	1364 (11.3)	167 (11.3)	0.97
Nausea	13,460	1648 (12.2)	1499 (12.5)	149 (10.2)	0.011
Vomiting	13,572	992 (7.3)	906 (7.5)	86 (5.8)	0.020
Diarrhea	13,617	3174 (23.3)	2885 (23.8)	289 (19.5)	< 0.001
Abdominal pain	13,566	867 (6.4)	776 (6.4)	91 (6.2)	0.70
Vital signs					
Confusion	13,576	1614 (11.9)	1451 (12.0)	163 (11.1)	0.33
Temperature					
Fever (> 38 °C)	13,254	2105 (15.9)	1911 (16.1)	194 (13.7)	0.019
Median (IQR) °C		37.0 (36.4–37.8)	37.0 (36.4–37.8)	36.8 (36.3–37.7)	< 0.001
Oxygen saturation %					
< 90%	13,316	2987 (22.4)	2783 (23.4)	204 (14.3)	< 0.001
Median (IQR) SatO2%		94 (91–97)	94 (91–96)	96 (93–97)	< 0.001
Tachypnea (> 20 breaths/min)	13,360	4,126 (30.9)	3772 (31.7)	354 (24.4)	< 0.001
Heart rate					
Tachycardia (> 100 beats/min)	13,254	2965 (22.4)	2681 (22.6)	284 (20.2)	0.035
Median (IQR)		87 (76–100)	87 (77–100)	85 (74–98)	< 0.001
SBP, median (IQR) mmHg	13,093	127 (114–141)	127 (114–141)	128 (115–140)	0.20
DBP, median (IQR) mmHg		73 (65–81)	73 (65–81)	74 (65–82)	0.67
Lung auscultation					
Crackles	13,357	7029 (52.6)	6434 (54.0)	595 (41.3)	< 0.001
Wheezing	13,353	811 (6.1)	725 (6.1)	86 (6.0)	0.86
Rhonchi	13,344	1442 (10.8)	1319 (11.1)	123 (8.5)	0.003

**Table 14:** Clinical outcomes in total population

On admission	Total (n = 13,932)	No. (%)	1 week after admission	Total (n = 13,932)	No. (%)	*p* value
Oxygen saturation %			Oxygen saturation %			
< 90%	13,493	3025 (22.4)	< 90%	11,467	1525 (13.3)	< 0.001
Median (IQR) SatO2%		94 (91–97)	Median (IQR) SatO2%		95 (93–97)	< 0.001
pH in arterial blood	7096	7.45 (7.41–7.48)	pH in arterial blood	2838	7.42 (7.37–7.46)	< 0.001
pCO2	7180	34 (31–39)	pCO2	2859	40 (35–46)	< 0.001
pO2	6827	66 (56–78)	pO2	2761	73 (60–91)	< 0.001
pO2/FiO2 mmHg	6540	289 (233–342)	pO2/FiO2 mmHg	2597	229 (120–328)	< 0.001

**Table 15:** Clinical outcomes among those who received antibiotics

On admission	Total (n = 13,736)	No. (%)	1 week after admission	No. (Total n = 13,736)	No. (%)	*p* value
Oxygen saturation %			Oxygen saturation %			
< 90%	13,316	2783 (23.4)	< 90%	11,339	1407 (13.8)	< 0.001
Median (IQR) SatO2%		94 (91–96)	Median (IQR) SatO2%		95 (93–97)	< 0.001
pH in arterial blood	6504	7.45 (7.41–7.48)	pH in arterial blood	2608	7.42 (7.38–7.46)	< 0.001
pCO2	6577	34 (31–39)	pCO2	2622	40 (35–46)	< 0.001
pO2	6288	66 (56–77)	pO2	2543	73 (60–90)	< 0.001
pO2/FiO2 mmHg	6026	288 (233–343)	pO2/FiO2 mmHg	2400	223 (119–325)	< 0.001

**Table 16:** Clinical outcomes among those who received macrolides

On admission	Total (n = 13,884)	No. (%)	1 week after admission	Total (n = 13,884)	No. (%)	*p* value
Oxygen saturation %			Oxygen saturation %			
< 90%	13,454	3,020 (22.5)	< 90%	11,439	914 (12.9)	< 0.001
Median (IQR) SatO2%		94 (91–96)	Median (IQR) SatO2%		95 (93–97)	< 0.001
pH in arterial blood	4721	7.45 (7.41–7.48)	pH in arterial blood	1997	7.43 (7.38–7.46)	< 0.001
pCO2	4785	34 (31–38)	pCO2	2017	40 (35–45)	< 0.001
pO2	4578	66 (56–77)	pO2	1957	74 (61–91)	< 0.001
pO2/FiO2 mmHg	4380	290 (235–343)	pO2/FiO2 mmHg	1844	223 (124–333)	< 0.001

### Laboratory findings

Laboratory findings showed an improvement in inflammatory parameters after one week of hospitalization with the exception of procalcitonin and ferritin, which showed no statistically significant changes in either group (general or those receiving antibiotics). Full data are shown in Tables 17 and 18. In the case of interleukin-6, there was a substantial decrease in the total study population after one week (median 30 pg/mL [IQR 11.4–65] vs. 16 pg/mL [IQR 4.8–53.6]; *p* < 0.05), but not in those who received antibiotics (median 31.6 pg/mL [IQR 11.9–66] vs. 16 pg/mL [IQR 4.9–56]; *p* = 0.068). Tables 19 and 20 show the changes at one week after admission in inflammatory parameters in patients who received antibiotics or macrolides.

**Table 17:** Laboratory findings in total population

On admission	No	Median (IQR)	1 week after admission	No	Median (IQR)	*p* value
Hemoglobin (g/dL)	13,622	13.9 (12.6–15)	Hemoglobin (g/dL)	12,646	13 (11.8–14.1)	< 0.001
Platelet count (× 10^6^/L)	13,636	190,000 (148,000–246,000)	Platelet count (× 10^6/L)	12,631	275,000 (199,000–371,000)	< 0.001
Leukocytes (× 10^6^/L)	13,620	6300 (4770–8,500)	Leukocytes (× 10^6/L)	12,644	6500 (4900–9000)	< 0.001
Neutrophils (× 10^6^/L)	13,558	4590 (3200–6700)	Neutrophils (× 10^6/L)	12,594	4325 (2900–6900)	0.025
Lymphocytes (× 10^6^/L)	13,613	940 (690–1300)	Lymphocytes (× 10^6/L)	12,626	1108 (700–1600)	< 0.001
C-reactive protein (mg/L)	13,127	59.1 (18.91–127)	C-reactive protein (mg/L)	12,248	23.5 (7–74.1)	< 0.001
Procalcitonin (ng/mL)	6452	0.1 (0.05–0.22)	Procalcitonin (ng/mL)	4907	0.09 (0.04–0.2)	0.061
Ferritin (mcg/mL)	5325	606 (291–1221)	Ferritin (mcg/mL)	6149	653 (337–1217)	0.36
Fibrinogen (mg/dL)	8789	610 (500–730)	Fibrinogen (mg/dL)	7852	573 (467–701)	< 0.001
Interleukin-6 [IL-6] (pg/mL)	1767	30 (11.36–65)	Interleukin-6 [IL-6] (pg/mL)	2074	16 (4.8–53.6)	0.045
Creatine kinase [CK] (U/L)	6844	91 (54–174)	Creatine kinase [CK] (U/L)	5775	54 (33–104)	< 0.001
Lactate dehydrogenase [LDH](mg/dL)	11,825	317 (245–428)	Lactate dehydrogenase [LDH] (mg/dL)	11,264	283 (219–402)	< 0.001
D-Dimer (ng/dL)	10,590	660 (372–1220)	D-Dimer (ng/dL)	9605	714 (384–1470)	< 0.001
Creatinine (mg/dL)	13,586	0.9 (0.73–1.16)	Creatinine (mg/dL)	12,599	0.82 (0.68–1.05)	< 0.001
Albumin (g/dL)	5717	3.8 (3.4–4.1)	Albumin (g/dL)	5358	3.4 (3.1–3.8)	< 0.001
Bilirubin (mg/dL)	10,296	0.5 (0.4–0.7)	Bilirubin (mg/dL)	9458	0.6 (0.4–0.89)	< 0.001
Alanine aminotransferase [GPT-ALT] (U/L)	12,786	29 (19–46)	Alanine aminotransferase [GPT-ALT] (U/L)	11,815	36 (22–64)	< 0.001
Aspartate Aminotransferase [GOT-AST] (U/L)	10,708	35 (25–52)	Aspartate Aminotransferase [GOT-AST] (U/L)	10,551	34 (23–53)	0.14

**Table 18:** Laboratory findings among those who received antibiotics

On admission	No	Median (IQR)	1 week after admission	No	Median (IQR)	p value
Hemoglobin (g/dL)	12,188	13.9 (12.6–15)	Hemoglobin (g/dL)	11,394	13 (11.8–14.1)	< 0.001
Platelet count (× 10^6^/L)	12,193	189,000 (148,000–246,000)	Platelet count (× 10^6^/L)	11,382	278,000 (200,000–374,000)	< 0.001
Leukocytes (× 10^6^/L)	12,186	6300 (4770–8500)	Leukocytes (× 10^6^/L)	11,394	6560 (4950–9100)	< 0.001
Neutrophils (× 10^6^/L)	12,130	4600 (3230–6750)	Neutrophils (× 10^6^/L)	11,352	4400 (2950–7070)	0.010
Lymphocytes (× 10^6^/L)	12,172	920 (680–1,300)	Lymphocytes (× 10^6^/L)	11,380	1100 (700–1590)	< 0.001
C-reactive protein (mg/L)	11,754	63 (21–131)	C-reactive protein (mg/L)	11,061	24.2 (7.1–77.5)	< 0.001
Procalcitonin (ng/mL)	5812	0.1 (0.06–0.23)	Procalcitonin (ng/mL)	4411	0.09 (0.05–0.21)	0.18
Ferritin (mcg/mL)	4821	627 (305–1,246)	Ferritin (mcg/mL)	5519	665 (346–1249)	0.20
Fibrinogen (mg/dL)	7867	611 (500–737)	Fibrinogen (mg/dL)	7021	573 (470–708)	< 0.001
Interleukin-6 [IL-6] (pg/mL)	1583	31.6 (11.9–66)	Interleukin-6 [IL-6] (pg/mL)	1856	16 (4.86–56)	0.068
Creatine kinase [CK] (U/L)	6262	92 (55–175)	Creatine kinase [CK] (U/L)	5309	54 (33–105)	< 0.001
Lactate dehydrogenase [LDH] (mg/dL)	10,618	320 (247–430)	Lactate dehydrogenase [LDH] (mg/dL)	10,151	285 (220–406)	< 0.001
D-Dimer (ng/dL)	9508	667 (380–1226)	D-Dimer (ng/dL)	8624	732 (395–1506)	< 0.001
Creatinine (mg/dL)	12,156	0.91 (0.70–1.21)	Creatinine (mg/dL)	11,366	0.83 (0.68–1.05)	< 0.001
Albumin (g/dL)	5199	3.8 (3.4–4.1)	Albumin -(g/dL)	4853	3.4 (3.1–3.8)	< 0.001
Bilirubin (mg/dL)	9259	0.5 (0.4–0.7)	Bilirubin (mg/dL)	8586	0.59 (0.40–0.87)	< 0.001
Alanine aminotransferase [GPT-ALT] (U/L)	11,515	29 (19–47)	Alanine aminotransferase [GPT-ALT] (U/L)	10,730	37 (22–66)	< 0.001
Aspartate Aminotransferase [GOT-AST] (U/L)	9519	36 (26–53)	Aspartate Aminotransferase [GOT-AST] (U/L)	9466	34 (23–54)	0.14

**Table 19:** Laboratory outcomes after using antibiotics

1 week after admission	No. (Total n = 13,932)	No. (%)	WITH antibiotics n = 12,238 (%)	WITHOUT antibiotics n = 1498 (%)	Odds ratio (95% CI)	*p* value
Anemia (Hb < 12 g/dL)	12,646	3760 (29.7)	3432 (30.1)	328 (26.2)	1.21 (1.06–1.39)	0.004
Thrombocytosis (Platelet count > 180)	12,631	10,191 (80.7)	9211 (80.9)	980 (78.5)	1.16 (1.01–1.34)	0.036
Leukocytosis (Leukocytes > 10,000)	12,644	2401 (19.0)	2255 (19.8)	146 (11.7)	1.87 (1.56–2.23)	< 0.001
Leukopenia (Leukocytes < 4000)		11,150 (88.2)	10,073 (88.4)	1,077 (86.2)	1.22 (1.03–1.45)	0.020
Lymphopenia (Lymphocytes < 1300)	12,626	4811 (38.1)	4211 (37.0)	600 (48.2)	0.63 (0.56–0.71)	< 0.001
Evolution of inflammatory parameters associated with covid-19						
C-reactive protein > 50 mg/L	12,248	4049 (33.1)	3761 (34.0)	288 (24.3)	1.61 (1.40–1.85)	< 0.001
Procalcitonin > 0.5 ng/mL	4907	606 (12.4)	575 (13.1)	31 (6.3)	2.25 (1.55–3.27)	< 0.001
Ferritin > 274 mcg/L	6149	4967 (80.8)	4506 (81.7)	461 (73.2)	1.63 (1.35–1.97)	< 0.001
Fibrinogen > 650 mg/dL	7852	2920 (37.2)	2602 (37.1)	318 (38.3)	0.95 (0.82–1.10)	0.50
CK > 200 U/L	5775	697 (12.1)	657 (12.4)	40 (8.6)	1.50 (1.08–2.10)	0.017
LDH > 300 U/L	11,264	5002 (44.4)	4593 (45.3)	409 (36.8)	1.42 (1.25–1.62)	< 0.001
IL-6 > 4.3 pg/mL	2074	1593 (76.8)	1428 (76.9)	165 (75.7)	1.07 (0.77–1.49)	0.68
D-Dimer > 250 ng/mL	9605	8367 (87.1)	7570 (87.8)	797 (81.2)	1.66 (1.40–1.97)	< 0.001

**Table 20:** Laboratory outcomes after using macrolides

1 week after admission	Total (n = 13,932)	No. (%)	With macrolides n = 8382 (%)	Without macrolides n = 5502 (%)	Odds ratio (95% CI)	No. Total (n = 13,932)
Anemia (Hb < 12 g/dL)	12,778	3800 (29.7)	2346 (29.8)	3439 (29.7)	1.00 (0.93–1.08)	0.97
Thrombocytosis (Platelet count > 180)	12,763	10,293 (80.7)	6504 (82.6)	3789 (77.6)	1.37 (1.25–1.50)	< 0.001
Leukocytosis (Leukocytes > 10,000)	12,776	2439 (19.1)	1611 (20.4)	828 (16.9)	1.26 (1.15–1.38)	< 0.001
Leukopenia (Leukocytes < 4000)		11,262 (88.2)	7008 (88.9)	4254 (87.0)	1.19 (1.07–1.33)	0.001
Lymphopenia (Lymphocytes < 1300)	12,758	4844 (38.0)	3006 (38.2)	1838 (37.6)	1.02 (0.95–1.10)	0.55
Evolution of inflammatory parameters associated with COVID-19						
C-reactive protein > 50 mg/L	12,375	4102 (33.2)	2418 (31.6)	1684 (35.7)	0.83 (0.77–0.90)	< 0.001
Procalcitonin > 0.5 ng/mL	4970	621 (12.5)	368 (12.6)	253 (12.3)	1.03 (0.86–1.22)	0.77
Ferritin > 274 mcg/L	6196	5010 (80.9)	3376 (80.7)	1634 (81.2)	0.97 (0.84–1.11)	0.62
Fibrinogen > 650 mg/dL	7927	2953 (37.3)	1622 (34.6)	1331 (41.0)	0.76 (0.69–0.83)	< 0.001
CK > 200 U/L	5828	706 (12.1)	426 (11.6)	280 (12.9)	0.89 (0.76–1.04)	0.15
LDH > 300 U/L	11,385	5065 (44.5)	3271 (45.9)	1794 (42.2)	1.16 (1.08–1.25)	< 0.001
IL-6 > 4.3 pg/mL	2097	1613 (76.9)	1124 (76.1)	489 (79)	0.84 (0.67–1.06)	0.14
D-dimer > 250 ng/mL	9698	8452 (87.2)	5462 (89.6)	2990 (83.0)	1.77 (1.57–1.99)	< 0.001

The decision to start antibiotics was determined by the presence of increased classical inflammatory markers such as C-reactive protein (OR 2.14, 95% CI 1.91–2.41; *p* < 0.05), procalcitonin (OR 1.73, 95% CI 1.28–2.35; *p* < 0.05), or leukocytosis (OR 1.18, 95% CI 1.01–1.38; *p* < 0.05). It was also determined by the presence of inflammatory markers associated with COVID-19, such as elevated lactate dehydrogenase (OR 1.30, 95% CI 1.16–1.47; *p* < 0.05), interleukin-6 (OR 1.73, 95% CI 1.16–2.59; *p* < 0.05), or ferritin levels (OR 1.93, 95% CI 1.59–2.35; *p* < 0.05) (Table 21). Table 22 shows the use of different antibiotics according to the previously described laboratory findings, with beta-lactams being the most used antibiotics among all groups.

**Table 21:** Decision to start antibiotic therapy based on initial inflammatory parameters

On admission	Total (n = 13,932)	No. (%)	With antibiotics n = 12,238 (%)	Without antibiotics n = 1498 (%)	Odds ratio (95% CI)	*p* value
Anemia (Hb < 12 g/dL)	13,622	2337 (17.2)	2082 (17.1)	255 (17.8)	0.95 (0.83–1.10)	0.51
Thrombocytosis (Platelet count > 180)	13,636	7533 (55.2)	6718 (55.1)	815 (56.5)	0.95 (0.85–1.06)	0.32
Leukocytosis (Leukocytes > 10.000	13,620	2077 (15.3)	1884 (15.5)	193 (13.5)	1.18 (1.01–1.38)	0.046
Leukopenia (Leukocytes < 4000)		1871 (13.7)	1658 (13.6)	213 (14.9)	1.11 (0.95–1.29)	0.19
Lymphopenia (Lymphocytes < 1300)	13,613	10,375 (76.2)	9401 (77.2)	974 (67.6)	0.61 (0.55–0.69)	< 0.001
C-reactive protein > 50 mg/L	13,127	7130 (54.3)	6615 (56.3)	515 (37.5)	2.14 (1.91–2.41)	< 0.001
Procalcitonin > 0.5 ng/mL	6452	764 (11.8)	716 (12.3)	48 (7.5)	1.73 (1.28–2.35)	< 0.001
Ferritin > 274 mcg/L	5325	4084 (76.7)	3758 (78.0)	326 (64.7)	1.93 (1.59–2.35)	< 0.001
Fibrinogen > 650 mg/dL	8789	3710 (42.2)	3336 (42.4)	374 (40.6)	1.08 (0.94–1.24)	0.28
CK > 200 U/L	6844	1436 (21.0)	1331 (21.3)	105 (18.0)	1.23 (0.98–1.53)	0.07
LDH > 300 U/L	11,825	6568 (55.5)	5969 (56.2)	599 (49.6)	1.30 (1.16–1.47)	< 0.001
IL-6 > 4.3 pg/mL	1767	1550 (87.7)	1400 (88.4)	150 (81.5)	1.73 (1.16–2.59)	0.007
D-dimer > 250 ng/mL	10,590	9226 (87.1)	8310 (87.4)	916 (84.7)	1.26 (1.05–1.50)	0.011

**Table 22:** Decision to start antibiotic therapy (and which one) based on initial inflammatory parameters

On admission	Beta-lactams		Macrolides		Quinolones	
	No (Total)	N. (%)	No (Total)	N. (%)	No (Total)	N. (%)
Anemia (Hb < 12 g/dL)	2368	1737 (73.4)	2364	1339 (56.6)	2346	330 (14.1)
Thrombocytosis (Platelet count > 180)	7627	5462 (71.6)	7619	4709 (61.8)	7556	977 (12.9)
Leukocytosis (Leukocytes > 10,000	2098	1602 (76.4)	2094	1222 (58.4)	2084	317 (15.2)
Leukopenia (Leukocytes < 4000)	1903	1333 (70.1)	1901	1114 (58.6)	1884	275 (14.6)
Lymphopenia (Lymphocytes < 1300)	10,500	7832 (74.6)	10,492	6432 (61.30)	10,412	1445 (13.9)
C-reactive protein > 50 mg/L	7214	5653 (78.4)	7212	4557 (63.2)	7154	1012 (14.2)
Procalcitonin > 0.5 ng/mL	776	651 (83.9)	774	445 (57.5)	768	110 (14.3)
Ferritin > 274 mcg/L	4118	3021 (73.4)	4117	2869 (69.7)	4099	390 (9.5)
Fibrinogen > 650 mg/dL	3749	2856 (76.2)	3751	2162 (57.6)	3720	432 (11.6)
CK > 200 U/L	1459	1151 (78.9)	1454	927 (63.8)	1444	199 (13.8)
LDH > 300 U/L	6647	5052 (76.0)	6641	4381 (66.0)	6588	824 (12.5)
IL-6 > 4.3 pg/mL	1563	1160 (74.2)	1564	1111 (71.0)	1556	110 (7.1)
D-dimer > 250 ng/mL	9313	6652 (71.4)	9318	6032 (64.7)	9247	1158 (12.5)

### Radiological findings

Pulmonary consolidation was present in 48.7% of patients and interstitial infiltrates in 62.6%. Involvement was mainly bilateral in both groups, particularly in those with interstitial infiltrates (bilateral involvement in 83.5% of patients with infiltrates). The presence of any kind of infiltrate was linked to antibiotic use (*p* < 0.05; see Table 23). Pleural effusion was present in less than 5% of patients and was not related to antibiotic use. A thoracic CT scan was performed in 774 patients (5.7%) and findings compatible with COVID-19 were observed in 88.7% of them; those with compatible findings had increased antibiotic use with (OR 3.53, 95% CI 1.85–6.73).

**Table 23:** Radiological outcomes after using antibiotics

	Total (n = 13,932)	No. (%)	With antibiotics n = 12,238 (%)	Without antibiotics n = 1498 (%)	Odds ratio (95% CI)	*p* value
At admission						
Condensation						
No	13,564	6962 (51.3)	6032 (49.7)	930 (65.2)	1. (ref)	–
Unilateral		2383 (17.6)	2206 (18.2)	177 (12.4)	1.92 (1.62–2.27)	< 0.001
Bilateral		4219 (31.1)	3899 (32.1)	320 (22.4)	1.88 (1.64–2.15)	< 0.001
Interstitial infiltrates						
No	13,572	5074 (37.4)	4388 (36.1)	686 (48.2)	1. (ref)	–
Unilateral		1399 (10.3)	1258 (10.4)	141 (9.9)	1.39 (1.15–1.69)	0.001
Bilateral		7099 (52.3)	6503 (53.5)	596 (41.9)	1.71 (1.52–1.92)	< 0.001
Pleural effusion						
No	13,565	12,942 (95.4)	11,573 (95.3)	1369 (96.1)	1. (ref)	–
Unilateral		411 (3.0)	377 (3.1)	34 (2.4)	1.31 (0.92–1.87)	0.14
Bilateral		212 (1.6)	191 (1.6)	21 (1.5)	1.08 (0.68–1.69)	0.75
Thoracic CT scan was performed	13,618	774 (5.7)	721 (5.9)	53 (3.6)	1.68 (1.26–2.23)	< 0.001
COVID-19 compatible findings on Thoracic CT	769	682 (88.7)	644 (89.9)	38 (71.7)	3.53 (1.85–6.73)	< 0.001
One week after admission						
Condensation						
No	10,132	4709 (46.5)	4123 (45.0)	586 (60.9)	1. (ref)	–
Unilateral		1406 (13.9)	1291 (14.1)	115 (12.0)	1.60 (1.29–1.97)	< 0.001
Bilateral		4017 (39.7)	3756 (41.0)	261 (27.1)	2.04 (1.76–2.38)	< 0.001
Interstitial infiltrates						
No	10,119	3562 (35.2)	3101 (33.9)	461 (48.1)	1. (ref)	–
Unilateral		753 (7.4)	685 (7.5)	68 (7.1)	1.50 (1.15–1.96)	0.003
Bilateral		5804 (57.4)	5374 (58.7)	430 (44.8)	1.86 (1.62–2.13)	< 0.001
Pleural effusion						
No	10,111	9647 (95.4)	8719 (95.3)	928 (96.9)	1. (ref)	–
Unilateral		302 (3.0)	282 (3.1)	20 (2.1)	1.50 (0.95–2.37)	0.08
Bilateral		162 (1.6)	152 (1.7)	10 (1.1)	1.62 (0.85–3.08)	0.14
Radiological worsening	10,154	4034 (39.7)	3774 (41.1)	260 (26.9)	1.89 (1.63–2.20)	< 0.001

Antibiotic use was also related to radiological worsening at one week after admission (OR 1.89; 95% CI 1.63–2.20; *p* < 0.001). Statistically significant differences were observed in the presence of pulmonary condensation and interstitial infiltrates at one week after admission in the group which received antibiotics. Changes were also noted in the presence of pleural effusion in the antibiotic group, but the difference was not significant. In the group which received macrolides, the percentage of patients with interstitial infiltrates remained the same, unlike other groups, as can be seen in Tables 24 and 25.

**Table 24:** Radiological evolution among those who used antibiotic therapy

	No. (Total = 12238)	No. (%)		No (Total = 12238)	No. (%)	*p* value
On admission			One week after admission			
Condensation	12,137	6105 (50.3)	Condensation	9170	5047 (55.0)	< 0.001
Interstitial infiltrates	12,149	7761 (63.9)	Interstitial infiltrates	9160	6059 (66.2)	0.001
Pleural effusion	12,141	568 (4.7)	Pleural effusion	9153	434 (4.7)	0.15

**Table 25:** Radiological evolution among those who used macrolides

	No. (Total = 8382)	No. (%)		No. (Total = 8382)	No. (%)	*p* value
On admission			One week after admission			
Condensation	8315	4301 (51.7)	Condensation	6390	3555 (55.6)	< 0.001
Interstitial infiltrates	8328	5440 (65.3)	Interstitial infiltrates	6386	4282 (67.1)	0.11
Pleural effusion	8318	360 (4.3)	Pleural effusion	6382	278 (4.4)	0.58

### Treatment and complications

Most patients received hydroxychloroquine (85.4%) and/or lopinavir/ritonavir (62.1%). In the antibiotic treatment group, more patients received hydroxychloroquine (87.3% vs. 70.1%; *p* < 0.001), lopinavir/ritonavir (62.1% vs. 55%; *p* < 0.001), and immunomodulators such as beta interferon, tocilizumab, anakinra, and systemic corticosteroids. The only therapy in which there were no differences between groups was immunoglobulins. All these data are shown in Table 26.

**Table 26:** Immunomodulatory therapies used among those who used antibiotic therapy

	Total (n = 13,932)	No. (%)	With antibiotics n = 12,238 (%)	Without antibiotics n = 1498 (%)	*p* value
Use of lopinavir/ritonavir	13,719	8414 (61.3)	7590 (62.1)	824 (55.0)	< 0.001
Use of hydroxychloroquine	13,727	11,727 (85.4)	10,677 (87.3)	1050 (70.1)	< 0.001
Use of beta-Interferon	13,662	1585 (11.6)	1488 (12.2)	97 (6.5)	< 0.001
Use of tocilizumab	13,703	1145 (8.4)	1106 (9.1)	39 (2.6)	< 0.001
Use of anakinra	13,604	76 (0.6)	76 (0.6)	0 (0)	< 0.001
Use of systemic corticosteroids	13,689	4738 (34.6)	4500 (36.9)	238 (16.0)	< 0.001
Use of immunoglobulin	13,483	62 (0.5)	60 (0.5)	2 (0.1)	0.06

Among the complications developed during hospitalization, higher mortality rates were observed in relation to several factors, including acute respiratory distress syndrome, acute heart failure, arrhythmias, acute kidney failure, shock, and sepsis. Bacterial pneumonia was found in 1481 patients (10.8%) and was more frequent among those who received antibiotics (OR 4.85, 95% CI 3.52–6.67; *p* < 0.001). Regarding respiratory support, oxygen via high-flow nasal cannula (OR 2.11, 95% CI 1.63–2.75; *p* < 0.001), non-invasive mechanical ventilation (OR 3.13, 95% CI 2.11–4.66; *p* < 0.001), and invasive mechanical ventilation (OR 4.21, 95% CI 2.84–6.25; *p* < 0.001) were used more often in the antibiotic group, as was prone positioning (OR 3.89, 95% CI 2.87–5.26; *p* < 0.001). A higher percentage of patients in the antibiotic group was transferred to intensive care units (ICU) compared to those who did not receive antibiotics (Table 27).

**Table 27:** Complications and clinical progress according to the  use of antibiotic therapy

	Total (n = 13,932)	No. (%)	With antibiotics n = 12,238 (%)	Without antibiotics n = 1498 (%)	Odds ratio (95% CI)	*p* value
Bacterial pneumonia	13,673	1481 (10.8)	1441 (11.8)	40 (2.7)	4.85 (3.52–6.67)	< 0.001
ARDS						
No	13,650	9190 (67.3)	7955 (65.4)	1235 (83.3)	1 (ref.)	–
Mild		1093 (8.0)	1033 (8.5)	60 (4.1)	2.67 (2.05–3.49)	< 0.001
Moderate		967 (7.1)	927 (7.6)	40 (2.7)	3.60 (2.61–4.97)	< 0.001
Severe		2400 (17.6)	2252 (18.5)	149 (10.0)	2.36 (1.98–2.82)	< 0.001
Acute heart failure	13,677	782 (5.7)	716 (5.9)	66 (4.4)	1.34 (1.04–1.74)	0.025
Arrhythmia	13,669	532 (3.9)	508 (4.2)	24 (1.6)	2.65 (1.75–4.01)	< 0.001
Epileptic seizures	13,680	81 (0.6)	74 (0.6)	7 (0.5)	1.29 (0.59–2.81)	0.52
Stroke	13,672	91 (0.7)	82 (0.7)	9 (0.6)	1.11 (0.56–2.22)	0.76
Acute kidney failure	13,673	1897 (13.9)	1757 (14.4)	140 (9.4)	1.62 (1.35–1.94)	< 0.001
Sepsis	13,667	822 (6.0)	780 (6.4)	42 (2.8)	2.35 (1.72–3.23)	< 0.001
Shock	13,656	605 (4.4)	582 (4.8)	23 (1.6)	3.19 (2.10–4.86)	< 0.001
Disseminated intravascular coagulation (DIC)	13,655	155 (1.1)	145 (1.2)	10 (0.7)	1.78 (0.94–3.39)	0.08
High-flow nasal cannula	13,635	1089 (8.0)	1027 (8.5)	62 (4.2)	2.11 (1.63–2.75)	< 0.001
Non-invasive mechanical ventilation	13,692	668 (4.9)	642 (5.3)	26 (1.7)	3.13 (2.11–4.66)	< 0.001
Invasive mechanical ventilation	13,696	874 (6.4)	848 (7.0)	26 (1.7)	4.21 (2.84–6.25)	< 0.001
Prone positioning	13,676	1361 (10.0)	1316 (10.8)	45 (3.0)	3.89 (2.87–5.26)	< 0.001
Intensive care unit admission	13,727	1095 (8.0)	1057 (8.6)	38 (2.5)	3.63 (2.62–5.04)	< 0.001
Death during hospitalization	13,736	2840 (20.7)	2597 (21.2)	243 (16.2)	1.39 (1.20–1.61)	< 0.001
Death during hospitalization and during readmission	13,549	2906 (21.5)	2653 (22.0)	253 (17.0)	1.37 (1.19–1.58)	< 0.001

The median length of hospital stay was eight days (IQR 5–13). The death rate in the group that received antibiotics was 21.2% and the death rate in the group that did not receive antibiotics was 16.2% (OR 1.40, 95% CI 1.21–1.62; *p* < 0.001). Ninety-four percent of the deaths were directly caused by COVID-19, with the remaining 6% occurring due to other reasons. Just 3.8% of patients were readmitted at a median time of 9 days after discharge (IQR 3–17); in 58.7% of these cases, readmission was unrelated to COVID-19. All these data are shown in Table 28.

**Table 28:** Resolution of covid-19 according to use of antibiotic therapy

	Total (n = 13,932)	No. (%)	With antibiotics n = 12,238 (%)	Without antibiotics n = 1498 (%)	Odds ratio (95% CI)	*p* value
Hospital stay in days, median (IQR)	13,736	8 (5–13)	9 (5–14)	7 (4–11)	0.99 (0.99–1)	0.16
Clinical outcomes						
Improvement: Discharge home	13,736	10,107 (73.6)	8938 (73.0)	1169 (78.0)	1 (ref.)	–
Discharge to other care centers		789 (5.7)	703 (5.7)	86 (5.7)	1.07 (0.85–1.35)	0.57
Death during hospitalization		2840 (20.7)	2597 (21.2)	243 (16.2)	1.40 (1.21–1.62)	< 0.001
Cause of death						
COVID-19	2796	2629 (94.0)	218 (91.6)	2411 (94.3)	1 (ref.)	–
Other causes		167 (6.0)	147 (5.8)	20 (8.4)	0.66 (0.41–1.08)	0.10
Hospital readmission	13,308	506 (3.8)	444 (3.8)	62 (4.2)	0.88 (0.67–1.16)	0.37
Days until readmission, median (IQR)	505	9 (3–17)	7 (3–16)	9 (3–18)	1.00 (0.98–1.02)	0.89
Cause of readmission						
COVID-19	504	208 (41.3)	176 (39.8)	32 (51.6)	1 (ref.)	–
Other causes		296 (58.7)	266 (60.2)	30 (48.4)	1.61 (0.95–2.75)	0.08
Death during hospitalization and during readmission	13,549	2906 (21.5)	2653 (22.0)	253 (17.0)	1.37 (1.19–1.58)	< 0.001

Tables 29 and 30 show the multivariate statistical analysis of the relationship between the use of antibiotic therapy and macrolides and mortality, adjusted for relevant clinical and analytical variables. We have chosen the procalcitonin level cut-off of 0.15 ng/mL as it has the best sensitivity and specificity profile after analysis using ROC curves. After statistical adjustment in the multivariate analysis, the use of antibiotic therapy is not statistically significantly related to a reduction in mortality (OR 1.20, 95% CI 0.94–1.53, p = 0.14). On the other hand, the use of azithromycin is associated with a lower odds of death (OR 0.64, 95% CI 0.56–0.73, p < 0.001).

**Table 29:** Use of antibiotic therapy and relationship to mortality (Multivariate analysis adjusted according to clinical variables)

	Odds ratio (95% CI)	*p* value
Use of antibiotic therapy	1.20 (0.94–1.53)	0.14
Age	1.08 (1.07–1.09)	< 0.001
Smoking status		
Never	–	1 (ref.)
Former	1.38 (1.19–1.59)	< 0.001
Current	1.63 (1.21–2.20)	0.001
Fever		
No (< 37 °C)	–	1 (ref.)
Low-grade fever (37–37.9 °C)	0.98 (0.80–1.20)	0.84
Fever (> 38 °C)	0.86 (0.72–1.03)	0.10
Shortness of breath	1.30 (1.13–1.49)	< 0.001
Oxygen saturation < 90%	2.21 (1.92–2.55)	< 0.001
Tachypnea	1.93 (1.68–2.21)	< 0.001
C-reactive protein (mg/L)	1.01 (1.01–1.02)	< 0.001
Procalcitonin (ng/mL) > 0.15	4.78 (3.81–5.99)	< 0.001
Use of systemic corticosteroids	1.50 (1.30–1.71)	< 0.001
Use of tocilizumab	1.90 (1.50–2.40)	< 0.001

**Table 30:** Use of macrolides and relationship to mortality (Multivariate analysis adjusted according to clinical variables)

	Odds ratio (95% CI)	*p* value
Use of macrolides	0.64 (0.56–0.73)	< 0.001
Age	1.08 (1.07–1.09)	< 0.001
Smoking status		
Never	–	1 (ref.)
Former	1.38 (1.19–1.59)	< 0.001
Current	1.62 (1.21–2.18)	0.001
Fever		
No (< 37 °C)	–	1 (ref.)
Low-grade fever (37–37.9 °C)	0.97 (0.79–1.18)	0.76
Fever (> 38 °C)	0.87 (0.73–1.04)	0.12
Shortness of breath	1.31 (1.14–1.51)	< 0.001
Oxygen saturation > 90%	0.45 (0.39–0.51)	< 0.001
Tachypnea	1.95 (1.70–2.24)	< 0.001
C-reactive protein (mg/L)	1.01 (1.00–1.01)	< 0.001
Procalcitonin (ng/mL) > 0.15	4.83 (3.86–6.04)	< 0.001
Use of systemic corticosteroids	1.60 (1.39–1.84)	< 0.001
Use of tocilizumab	1.89 (1.49–2.39)	< 0.001

## Discussion

Since the start of the COVID-19 pandemic, efforts have been made to show the role that antibiotics associated with antivirals, anti-inflammatories, and other immunomodulatory drugs may play in order to define an effective therapy against COVID-19.

Some authors think that the difficulty in finding antiviral treatments with proven efficacy along with the anxiety and uncertainty that this generates in physicians has likely led to the uncontrolled prescription of antibiotic therapy in patients worldwide [26]. Indeed, emerging data show that more than 90% of COVID-19 patients receive antibacterial drugs [27, 28].

In the Chinese city of Wuhan, where the pandemic started, most patients with COVID-19 seem to have received empiric antibiotic therapy, mostly respiratory fluoroquinolones [29]. The use of antifungal drugs and corticosteroids was more limited. Similar data are described in other studies in China, revealing use of antibiotic therapy in more than half of hospitalized patients [30–33].

In the United States of America, the strategy for empiric antibiotic therapy has been along these same lines. More prevalent antibiotic use was revealed in ICU patients, where 94.9% (224/236) were on antibiotics [34]. In another series in Detroit, antibiotic use in 69.2% (148 of 214 patients) of patients admitted to the conventional ward was documented; their study population had baseline characteristics that were similar to ours [35].

Langford et al. have conducted a rapid systematic review that determined that the majority of patients with COVID-19 received antibiotics (71.8%, 95% CI 56.1–87.7). The most common were broad-spectrum antibiotics, with fluoroquinolones and third-generation cephalosporins representing 74% of the antibiotics prescribed [36].

The work by Beovic et al. consisted of a survey aimed at doctors in Europe. As was the case in Asia and America, the study revealed indiscriminate use of broad-spectrum antibiotic therapy. In particular, the study highlights that Spain is one of the countries with the highest rates of antibiotic use—only 22.7% of patients with COVID-19 in the conventional ward were not routinely prescribed antibiotics—behind only Italy (18.2%) and Turkey (19.6%) [37].

### What causes the indiscriminate use of empiric antibiotic therapy in COVID-19 patients?

Antibiotics are usually prescribed in light of the possibility that these patients may have a bacterial infection associated with the ailment that is either concomitant with the initial viral infection or in relation to an extended hospital stay [38, 39].

It is known that bacteria (especially *Streptococcus pneumoniae* and *Staphylococcus aureus*) as well as other viral or fungal co-infections are frequent complications that occur in seasonal influenza outbreaks which contribute to increased morbidity and mortality in these patients [40–42]. Previous studies have documented that fatality associated with viral pneumonias may be influenced by multiple factors, one of the most prominent being bacterial co-infection [43, 44]. In fact, most bacterial co-infections linked to a primary viral infection are seen in influenza cases [45]. Several studies from the USA and Australia found that in the 2009 H1N1 flu pandemic, 4–33% of patients hospitalized due to that disease had bacterial pneumonia [45–49].

Co-infection by bacteria and viruses in respiratory infections is not only restricted to influenza. Similar conditions have also been reported in other respiratory viruses such as the parainfluenza virus, respiratory syncytial virus, adenovirus, rhinovirus, human metapneumovirus, and even in pathogens similar to SARS-CoV-2 such as SARS (Severe Acute respiratory syndrome) and MERS (Middle-East respiratory syndrome) [50–53].

Nevertheless, the current evidence on SARS-CoV-2 indicates that the risk of bacterial co-infection upon admission is minimal, though risk increases progressively during hospitalization and critical patients are at highest risk [54]. In several studies conducted in China and Italy, rates of bacterial infection of < 10% were found [55, 57]. In a meta-analysis by Langford et al., in which a total of 1308 publications were reviewed with 24 studies included in the final statistical analysis, the presence of bacterial infection was assessed in 3338 patients and found in 281 of them (8.4%) [36].

Although the actual prevalence of bacterial infection in patients with SARS-CoV-2 pneumonia has not been fully demonstrated and further studies are needed, several clinical guidelines advocate for using empiric antibiotic therapy in patients with COVID-19, especially in critically ill patients [58, 59]. Many guidance documents recommend antibiotic treatment for patients with COVID-19 and ‘pneumonia’ [60].

In the survey of European doctors carried out by Beovic et al., nearly two-thirds of participants reported that they did indeed have local guidelines regarding antibiotic use in patients with COVID-19 [37], but more often than not, they followed their hospital’s community-acquired pneumonia guidelines [15]. Most professionals opted for coverage of pathogens that cause atypical pneumonia. However, these guidelines appear to be grounded in the experience gained in studies of co-infection in patients with influenza, in which the majority were caused by *Streptococcus pneumoniae* and *Staphylococcus aureus* [61]. In light of this, several authors recommend that if antibiotics are considered, a beta-lactam providing coverage for *S. pneumoniae* ± methicillin-susceptible *S. aureus* should be the first [26]. In contrast, other researchers, such as the Greek group Karampela et al., recommend fluoroquinolones when starting antibiotic therapy [19] based on the fact that these quinoline derivatives (the prodrome of chloroquine) appear to have an ability to suppress SARS-CoV-2 replication by exhibiting a stronger capacity for binding to its main protease than chloroquine and antiretrovirals such as nelfinavir [62, 63].

The Spanish group García-Vidal et al. aimed to determine the epidemiology, impact, and outcomes of co-infections in a cohort of 989 consecutive patients hospitalized with COVID-19 [64]. A total of 88 co-infections were documented in 72 patients (7.3%). They recommend using empiric antibiotic therapy only in COVID-19 patients who had a chest x-ray suggestive of associated bacterial pneumonia, those who required admission to the ICU, and those who were previously immunosuppressed.

We conclude that the use of antibiotic therapy has been unreasonable given that nearly 90% of patients admitted to internal medicine departments received them empirically (12,238 of 13,932 patients, 87.8%). The most used antibiotics were beta-lactams (72.0%), macrolides (60.2%), and fluoroquinolones (13.3%), which is in line with the available data from the rest of EU (European Union). This pattern of use can plausibly be attributed to the fact that empiric use of third-generation cephalosporins together with azithromycin was included in most hospital protocols in the first months of the pandemic.

The vast majority of our patients had community acquisition of COVID-19; only 6.6% acquired the infection in a hospital. Also of note is the fact that infection in nursing homes occurred in < 10% of cases. Antibiotic use, and specifically macrolide use, correlated to where the infection was contracted: their use was more common among those with community-acquired infection and less common among those who contracted the disease in nursing homes or the hospital.

### For which patient profiles should antibiotic therapy be considered?

There appears to be broad consensus on initiating antibiotic treatment in all severely ill patients who require direct admission to the ICU upon arrival at the hospital [24, 59]. However, most authors coincide in highlighting the difficulty of distinguishing SARS-CoV-2-related pneumonia versus atypical pneumonia or nosocomial ventilator-associated pneumonia in COVID-19 patients based on symptoms alone, given that all present with similar signs and symptoms consisting of fever, dry cough, dyspnea, and bilateral involvement on imaging tests. For this reason, they argue that physicians should avail themselves of analytical results when making a decision on whether or not to use antibiotics [10, 26, 32, 39, 65].

Indeed, this is precisely what is being done on a daily basis at the patient's bedside. In research by Beovic et al., physicians indicated that patients’ clinical presentation was the most significant factor when considering starting antibiotic therapy, followed by elevated inflammatory parameters on laboratory tests and radiological findings of pneumonia. Among the analytical results, the most relevant were elevated procalcitonin levels, the neutrophil count, the degree of leukocytosis, and elevated C-reactive protein (CRP) levels [37].

In our population, we found that the most critical clinical information used when determining whether to begin empiric antibiotic therapy in COVID-19 patients was symptoms such as the presence of fever, dyspnea, and cough (especially productive) were similar to what was reported in the literature. Other symptoms that are more closely related to viral infections, such as arthralgia; fatigue; anorexia; and gastrointestinal symptoms such as nausea, vomiting, and diarrhea, are also associated with greater use of antibiotics. On the other hand, the presence of anosmia, ageusia, headache, or abdominal pain did not seem to have an influence on antibiotic use. The most relevant data on the physical examination were those that reflected more severe disease: oxygen saturation < 90%, tachypnea, and tachycardia. Furthermore, patients who had crackles and rhonchi were more likely to receive antibiotics, findings that were statistically significant; those with wheezing were also more likely to receive antibiotics, but this finding was not significant.

In regard to patients’ previous treatment, it would be logical to believe that those on immunosuppressive treatments would have received antibiotics at a higher rate, but no differences were observed in antibiotic use according to prior immunosuppressive treatment and as such, these drugs were not found to be critical in decision-making regarding use of antibiotics. Only those taking hydroxychloroquine were observed to have received antibiotics more often. Among the group that received macrolides, antibiotics were used less frequently among those being treated with systemic corticosteroids or biological therapies.

Concerning the influence of analytical parameters on the decision to start antibiotic therapy, the results are clear: the elevation of inflammatory parameters such as CRP, procalcitonin, ferritin, LDH (lactate deshidrogenase), and D-dimer have proven to be the most relevant factors in the decision to begin antibiotic treatment, as indicated in previous works. Leukocytosis, interpreted as a sign of risk of bacterial infection, was related to greater use of antibiotics whereas lymphopenia, more often linked with viral symptoms, was inversely related to the use of antibiotics.

Rapid characterization of co-infection is essential in order to properly guide antibiotic management and could help to save lives during the pandemic [57]. Huttner et al. recommended that in cases in which antibiotics are to be started, microbiological samples such as a urinary antigen test for Legionella and blood cultures, should be obtained beforehand in order to diagnose the co-infection [26]. Mirzaei et al. also advocated for a proper diagnosis, noting the importance of a broad-spectrum molecular diagnostic panel for rapid detection of the most common respiratory pathogens [39].

We believe that actively searching for possible bacterial co-infection and early diagnosis are aspects of caring for COVID-19 patients that must be improved. A urinary antigen test for Legionella and *S. pneumoniae* was performed in less than half of patients and though there was a very small rate of positive tests (1.5%), mortality was found to be higher among those who did test positive. Antibiotic therapy was used less frequently in patients who did not have a urinary antigen test, but this is likely due to little suspicion of initial bacterial co-infection that resulted in these patients not being prescribed antibiotics. Unfortunately, we do not have information on blood or sputum cultures; this is a possible area of future research.

### Comparisons to other studies

Other retrospective case series similar to ours found. A work by Argenziano et al. analyzed the first 1000 patients hospitalized for COVID-19 in the New York City region [34]. The mean age was 63.0 years and predominantly male (57.5%). There were high rates of baseline comorbidities, the most common of which were hypertension and diabetes mellitus. The most common symptoms on admission were dry cough (73.2%), fever (72.8%), and dyspnea (63.1%). They also report that patients with marked elevation of inflammatory parameters (CRP, ESR -erythrocyte sedimentation rate-, D-dimer, ferritin, and LDH) were those who most frequently required transfer to the ICU. In this series, 21.1% of patients across all levels of care died (14% when only considering patients in conventional wards).

Suleyman et al., in a series of 463 cases in Detroit, studied a population with a mean age of 57.5 years that was predominantly female (55.9%) and African American (72.1%) [35]. Virtually all patients (94%) had at least one comorbidity, the most common of which were hypertension (63.7%), chronic kidney disease (39.3%), and diabetes (38.4%). They had similar symptoms upon admission as those in our study: cough (74.9%), fever (68.0%) and dyspnea (60.9%). A higher death rate (20%) was observed in this work compared to previous studies, with male gender and age (over 60 years) shown to be the most relevant risk factors.

In Liang et al.’s work on a cohort of 1590 cases in China, a younger mean age was observed: 48.9 years. Nine hundred and four (57.3%) patients were male and 399 (25.1%) had comorbidities, including hypertension (16.9%), diabetes (8.2%), and cardiovascular disease (3.7%). Fever (88.0%), dry cough (70.2%), fatigue (42.8%), productive cough (36.0%) and shortness of breath (20.8%) were the most common symptoms [66]. The overall rates of severe cases and fatality was 16.0% and 3.2%, respectively.

Our cohort of patients had a mean age of 69.0 years, which is older than in the mentioned studies; the mean age was even higher among the group which received antibiotics. One finding that merits mention is that the use of antibiotic therapy was lower in the group of patients over 80 years of age and in frail patients, defined as those with dementia, neurodegenerative diseases, or a high degree of dependence. In regard to the rest of the demographic data and comorbidities, no differences were noted in terms of use of antibiotic therapy except for among men and those with cardiovascular risk factors (hypertension, dyslipidemia, and diabetes), in which there was a higher percentage of use.

We found higher death rates in our patient sample compared to previous research. The overall fatality rate was 20.7% (2840 of 13,736 patients). A striking finding was the higher death rate among those who received any antibiotic (OR 1.39, 95% CI 1.20–1.61) except macrolides, in which there was a higher survival rate (OR 0.70, 95% CI 0.64–0.76; *p* < 0.001). Even considering that use of antibiotic therapy was lower in patients who a priori had a higher risk of dying, namely older or more frail patients, the relationship between antibiotic therapy and fatality persisted even after controlling for these confounding favors on the logistic regression (OR 1.52, 95% CI 1.29–1.80).

In terms of the clinical progress of patients in whom antibiotics were used, improvement was observed in most inflammatory parameters, though there was radiological worsening, with an increase in the proportion of patients with consolidation or interstitial infiltrates. Moreover, antibiotics did not diminish the risk of developing bacterial co-infections among hospitalized patients, as bacterial pneumonia was found in 1481 patients (10.8%) and it was more frequent in those who received antibiotics.

Other complications occurred more frequently during hospitalization, including acute respiratory distress syndrome, acute cardiac failure, arrhythmias, acute renal failure, shock or sepsis, and increased demand for respiratory support (oxygen via high-flow nasal cannula, non-invasive mechanical ventilation, invasive mechanical ventilation, and prone positioning). A higher percentage of patients in the group that received antibiotics required ICU admission. These findings could possibly be explained by the fact that use of empiric antibiotic therapy was widely generalized; its use was only limited among patients who were very frail (and thus not candidates for invasive measures) or, on the contrary, among patients with very mild symptoms.

### The role of macrolides

Macrolides have been proposed as a possible treatment for severe acute respiratory distress syndrome caused by COVID-19 since the first months of the pandemic [21, 23]. These bactericidal antibiotics are widely used in habitual clinical practice against gram positive and atypical bacteria species that are usually associated with respiratory tract infections. The antiviral effects of macrolides have attracted considerable attention. Their ability to modulate the immune response and decrease the production of inflammatory cytokines makes them a very interesting tool for battling respiratory viral infections. The efficacy of macrolides in the treatment of other respiratory viruses such as rhinovirus, respiratory syncytial virus, and influenza has long been established [22, 25]. In addition to the aforementioned respiratory viruses, azithromycin has also been reported to inhibit Zika virus [24].

In terms of COVID-19, azithromycin was one of the drugs included in the large adaptive RECOVERY trial [67]. Based on preclinical and clinical evidence and some preliminary results in COVID-19 patients, azithromycin could have potential in the fight against this new disease [68].

In a clinical trial led by Gautret et al. in France, a combination of hydroxychloroquine and azithromycin was shown to be effective against COVID-19 [69]. Treatment efficacy was compared in 36 patients divided into three groups: six patients were treated with hydroxychloroquine combined with azithromycin, 14 with hydroxychloroquine in monotherapy, and 16 with a placebo. The results showed that by the sixth day of treatment, all patients in the HCQ + AZM group had no detectable virus in nasopharyngeal exudate samples compared to 57.1% of the HCQ group and 12.5% of the control group (*p* < 0.001).

In our study, we found a favorable outcome with the use of macrolides compared to other antibiotics. As we have highlighted, the mortality rate was lower in the macrolides group (unlike with other antibiotics) and indeed, the survival ratio was higher among patients who received them, a finding that was statistically significant (OR 0.70, 95% CI 0.64–0.76). Patients in whom macrolides were used were younger than those who received other antibiotics (68 years vs. 71 years). In order to control for possible confounding variables, a multivariate analysis was conducted that showed that the use of macrolides in our population continued to be linked to a lower mortality rate (OR 0.80, 95% CI 0.73–0.88).

Huttner et al. consider that macrolides and quinolones should be avoided due to the risk of cardiotoxicity [37]. Along these lines, a lower rate of use of azithromycin was observed among patients with previous heart disease in our study.

The risk of a rise in multidrug-resistant germs due to indiscriminate antibiotic use has been described in the literature [70–72]. The exact incidence of bacterial superinfections in COVID-19 patients is still not entirely clear and the incidence seems to be much lower than in severe influenza [8]. We agree with many other authors that establishing clear criteria for initiating antibiotic therapy in COVID-19 patients is essential in order to prevent the consequences of inappropriate prescribing [26, 37, 64]. We must be aware that a potential consequence of the COVID-19 pandemic is the long-term propagation of antimicrobial resistance resulting from increased patient exposure to antimicrobials that are often suboptimally or inappropriately used [72, 73]. This rapid growth in antibiotic prescribing can exercise a strong selective pressure on bacterial pathogens to develop resistance, leading to increased incidence of drug-resistant bacterial infections in the years following the COVID-19 pandemic. It has been calculated that ten million people could die from antibiotic-resistant bacterial infections each year by 2050 [39].

Recently, a group of members of ESCMID’s Study Group for Antimicrobial Stewardship (ESGAP) published a paper warning against non-critical use of antibiotics in COVID-19 patients along with some practical recommendations. Huttner et al. indicate that we should periodically reevaluate the suitability of our prescription and discontinue it as soon as possible when there is low suspicion of bacterial infection. In the event its continued use is warranted, switch to oral therapy early and give short cycles of five days [26]. It is important to educate healthcare providers in antimicrobial stewardship to prevent the consequences of excessive antimicrobial use such as toxicities, selection for opportunistic pathogens such as *Clostridioides difficile* (coinfection with SARS-CoV-2 results in a worsening of outcomes) and antimicrobial resistance [74, 75].

## Conclusion

In this multicenter, retrospective study, the overall percentage of bacterial co-infection among patients with COVID-19 was low, but the use of antibiotics was high. There is insufficient evidence to support widespread use of empiric antibiotics in patients hospitalized for COVID-19. The majority of these patients may not require empiric antibacterial treatment and, if it is needed, there is promising evidence regarding the use of azithromycin as a potential treatment for COVID-19. However, more structured studies must be carried out in this regard.

Our outcomes provide evidence against the use of antibiotic therapy in most patients hospitalized for COVID-19 since it has not been proven to reduce the mortality rate of these patients. We recommend against routinely prescribing antibiotics to all hospitalized patients with COVID-19.

### Future lines of research

There is a lack of data on bacterial co-infections in COVID-19 patients. This information is essential for determining the role of empiric antimicrobial therapy and antibiotic stewardship strategies. Biomarkers (CRP, procalcitonin) may play a role in deciding which patients should not receive antibiotics, but further investigation is required.

Prospective clinical studies on antibiotic prescription and systematic analyses of COVID-19 patients diagnosed with bacterial co-infection must conducted in order to evaluate the influence of current and future viral pandemics on antimicrobial resistance and the development of superinfections. This line of research is critical for avoiding unintended consequences resulting in broad antimicrobial resistance in the near future.

Lastly, standard guidelines for the administration of the antibiotics must be established.

## Supplementary Information

**Additional file 1.** List of the SEMI-COVID-19 Network members.

## Data Availability

All data generated or analysed during this study are included in this published article and its Additional files.

## Abbreviations

SARS-CoV-2: Severe acute respiratory syndrome coronavirus 2
COVID-19: Coronavirus disease 2019
WHO: World Health Organization
FDA: Food and drugs administration
SEMI: Spanish society of internal medicine
Rt-PCR: Real time transcription polimerase chain reaction
IQR: Interquartile range
ANOVA: Analysis of variance
OR: Odds ratio
CI: Confidence interval
ICU: Intensive care units
CT: Computerized tomography
SARS: Severe acute respiratory syndrome
MERS: Middle-East respiratory syndrome
EU: European Union
CRP: C-reactive protein
ESR: Erythrocyte sedimentation rate
LDH: Lactate deshidrogenase
CK: Creatinine kinase
ESCMID: European Society for Clinical Microbiology and Infectious Diseases
ESGAP: Study Group for Antimicrobial Stewardship
AIDS: Acquired immunodeficiency syndrome
HAART: Highly antiretroviral therapy
